# herbUA Collectors: An open-source framework for online publication of the herbarium collector-centric data

**DOI:** 10.3897/BDJ.14.e185211

**Published:** 2026-03-16

**Authors:** Andriy Novikov

**Affiliations:** 1 State Museum of Natural History of the NAS of Ukraine, Lviv, Ukraine State Museum of Natural History of the NAS of Ukraine Lviv Ukraine

**Keywords:** virtual herbarium, digitisation, collectors, data visualisation, WordPress

## Abstract

**Background:**

The digitisation of herbarium collections is typically aimed at mobilising and distributing data about the specimens, while often omitting data about the individuals who contributed to the formation of these collections. Despite the obvious need to create databases of herbarium collectors, as well as to structure and standardise the data about them, there is currently no consolidated view on this regard. Many efforts have been made to develop various collector-centric databases, but there is no simple and quick way to present such data online by self-running herbaria or small initiatives. Therefore, here I introduce a framework that allows for easy configuration of WordPress to display data about herbarium collectors.

**New information:**

This article describes the herbUA Collectors repo, allowing for the configuration of a standard WordPress installation for: (a) filling and online publishing the database of the herbarium collectors; (b) representing records about each collector as a single web page; (c) providing an interactive map search facility; (d) visualising the basic statistics on the database.

## Introduction

Digitisation is a crucial stage in the modern management of herbaria, aimed at both data mobilisation and digital image processing, followed by the publication and long-term storage of the resulting data. Over the last few decades, it has rapidly evolved from a relatively simple database to a complex and integrative approach, resulting in well-structured and annotated data, which allows for the remote manipulation of digital specimens through the internet (Davis 2023, Paton et al. 2025). Such remote access to digital specimens is realised through virtual herbaria (e.g. JACQ (2026), Open Herbarium (2026), Botanical Collections (2026)) or different data aggregators (e.g. GBIF (2026), Europeana (2026)). The online publication of digitised materials enables the realisation of basic (e.g. specimen identification, mapping or checklist creation) and advanced (e.g. specimen annotation, morphometrics or distribution modelling) research tasks without direct access to the specimens (Soltis 2017, Nieva de la Hidalga et al. 2020).

In most cases, herbarium digitisation is specimen-centric, with less attention paid to collectors' bibliographic data. Herbarium digitisation initiatives are rightly centred on individual specimens. This focus reflects their core mission to mobilise the data about specimens. However, to fully realise the scientific potential of digitisation initiatives, it is crucial that they are supported by comprehensive and structured collector-centric metadata. In particular, the information about collectors can be evidently useful and significantly simplify the label trascription (see Ewan (1969) for examples). Information about years of life and collecting activity, along with data on collecting regions, can aid in the detection of uncertain specimens (e.g. those with uncertainly indicated toponyms or collecting dates). In turn, this allows more accurate dating and georeferencing of the specimens. Another benefit is gained from gathering collectors’ handwriting and signature samples. Ambiguous handwriting can lead to the incorrect transcription of the collection locality or date (Ewan 1969, Willis et al. 2017). Scripting expertise can help attribute uncertain specimens (e.g. those with unclearly indicated collectors or without such information) to a specific collector, thereby clarifying their origin and collecting period. Moreover, handwriting samples can be beneficial for automatic text recognition and training AI-based annotation models (e.g. for DiSSCover (2026) infrastructure), as handwritten data remains a challenge for them (Drinkwater et al. 2014, Thompson et al. 2023, Guralnick et al. 2024).

Many efforts have been made to develop various collector-centric databases, with their own vision and natural limitations. These databases significantly simplify the work with the natural history collections, including herbaria and deserve praise. This paper and the herbUA framework are not intended to replace them or to criticise, but rather to propose one more option for standardising and publishing collector-centric data online. Below, I briefly overview the principal existing initiatives, their features and the aspects that led me to create the herbUA framework. First of all, Bionomia (2026) is amongst the unique online platforms that realise the collector-centric aspect of digitised collections, which harvests primary data from GBIF (2026). Synchronisation with Wikidata (2026) and ORCID (2026) enables it to complete the contributors' (collectors or identifiers) profiles with brief biographical data, including their names and spelling variants, birth dates and, in some cases, signature samples. Based on the data harvested from GBIF, Bionomia also provides information about the years and regions of activity, as well as the taxonomic coverage of the contributors. It also allows authorised users to review and correct the records. Of course, Bionomia has its own limitations predicted by its scope. It does not allow linkage with other virtual collections and authority databases (e.g. IPNI (2026) or VIAF (2026)). It is also not intended for building virtual collections or separate databases of collectors, nor for direct editing of collector profiles. The principal scope of IPNI is to represent standardised nomenclatural data on plant names. Within this framework, it provides extensive information about botanists who contributed to naming the plants. In particular, it provides standard forms for authors, listing their years of living and indicates their area and geographic interests. However, due to its strict scope, this database does not cover all herbarium collectors. Another global initiative, the Harvard Index of Botanists (2026), provides similar information regarding botanists worldwide, including their geographical interests, activity years, taxonomic coverage etc. However, it remains conservative and has no graphical visualisation (e.g. interactive map). Harvard Index of Botanists (2026) is also not interlinked with other data sources and, unlike Bionomia, does not allow tracking of the specimens. Regional initiatives similar to Harvard Index of Botanists include Index Personarum et Institutionum in der Datenbank der CNSflora (2026), Botanická Bibliografická Databáze (2026), Osobnosti Botaniky na Slovensku (2026), Homo Botanicus (2026) and Die Herbonauten (2026). There are many other global or regional initiatives that, in some way, allow us to clarify and track the data regarding the herbarium collectors like Zobodat (2026), Österreichisches Biographisches Lexikon (2026), Biografický Slovník Českých Zemí (2026) or VIAF (2026). Special attention is due to the database IndExs (Triebel and Scholz 2026), which contains data on world herbarium exsiccatae and exsiccata-like specimen series, as well as their editors. Although these databases are not strictly focused on the herbarium collectors, they served as a vital additional source of data.

At the same time, many herbaria prefer to maintain their own virtual environments adapted for their own purposes or collaborate within extended initiatives like JACQ (2026) or Reflora (2026). Some virtual herbaria are constructed as stand-alone frameworks, while others use the Symbiota (2026) environment. For example, the herbaria of Warsaw University (Zielnik Wydziału Biologii Uniwersytetu Warszawskiego 2026), University of Coimbra (Willkomm Herbarium 2026), Zurich University (Zürcher Herbarien 2026), Strasbourg University (Herbier de l’Université de Strasbourg 2026) and Göttingen University (Georg-August-Universität Göttingen 2026) have their own indices of collectors. Interesting and one of the earliest examples of the online realisation of the index of herbarium collectors is the Cyclopaedia of Malesian Collectors (van Steenis Kruseman 2017). Besides the brief biography and collecting activity information, it also includes details about the collectors' careers. This database also allows navigation through different herbaria to which the listed collectors contributed.

Mentioned collectors' lists differ in their development principles, completeness and visualisation. They have various integrations with other databases and usually do not offer downloading functionalities. However, they all help to extend knowledge regarding herbarium collectors, clarify their biographical details and simplify work with herbarium collections. Unfortunately, there was no open framework allowing for the easy construction and online representation, which could extend the number of collectors' databases. Therefore, in 2025, I developed the herbUA Collectors framework, which is presented here and could serve as a supportive to the databases mentioned above.

## Project description

### Title

herbUA Collectors

### Design description

The herbUA Collectors is a WordPress extension comprising three custom plugins and one child theme. With the help of three other official WordPress plugins, i.e. Advanced Custom Fields (WP Engine 2026), Custom Post Type UI (Webdevstudios 2026) and WP All Export (Soflyy 2026), it allows adapting the WordPress for maintaining and publishing the database of herbarium collectors. WordPress has been chosen as it is free and is one of the most popular content management systems. All mentioned plugins have free versions to use and are expected to be maintained continuously due to their popularity too.

The herbUA Collectors extension has been developed within the project of the digitisation of the Herbarium of the State Museum of Natural History of the NAS of Ukraine (Lviv, Ukraine). This Herbarium contains ca. 147,000 specimens, most of which (over 120,000 specimens) represent vascular plants (Novikov and Nachychko 2025). It is expected that this Herbarium comprises nearly 500 collectors' names, 370 of which were confirmed during the digitisation. The online database Collectors of Ukrainian Herbaria was launched in 2025 and currently contains 154 profiles of collectors available online (Novikov 2025). This database provides data on herbarium collectors who worked in Ukraine and adjacent regions. The biographies of such collectors are often poorly known, published in Cyrillic sources or not available online at all. It also benefits from providing the photograps of the labels with handwriting and/or signatures of these collectors. In its logic, Collectors of Ukrainian Herbaria aims to complement similar collector-centric databases. Although this database is not completed yet, it can be used as a demonstration version of the presented extension facilities.

**External plugins**

The three plugins listed below can be downloaded through the WordPress interface using the standard procedure (Dashboard → Plugins → Add plugins → Search Plugins [look for listed plugins’ names] → Instal Plugin). After that, they must be activated (Dashboard → Plugins → Installed plugins → [choose required plugin] → Activate).

**Figure 1.**
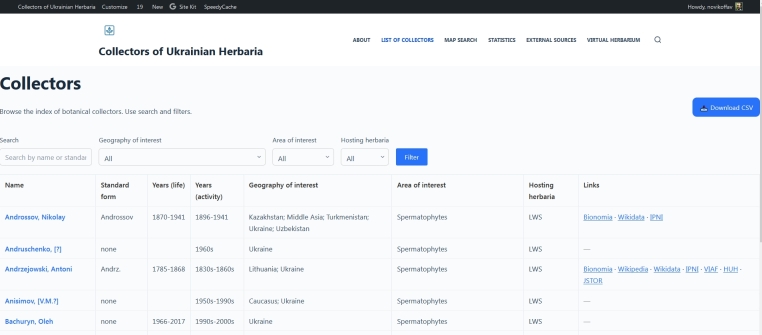
The page of the Collectors of Ukrainian Herbaria representing the main database, searching and filtering options.

The plugin *Custom Post Type UI (CPT UI)* (Webdevstudios 2026) is used for creating and setting up the post template ‘collector’. This template serves as the initial point of creation for the database; therefore, its set-up is a crucial step. All custom fields are later applied to this post template. The Custom Post Type UI plugin is also applied to set the taxonomies ‘geography’, ‘area’ and ‘herbarium’. The taxonomy ‘geography’ is labile and does not follow any scheme or controlled vocabulary. However, only terms that match the built-in country list in "MapSVG" will be displayed on the interactive map. In the interactive map plugin, a built-in normaliser of the country name spelling variants is applied, so variations and synonyms also work. The taxonomy ‘geography’ is used to indicate geographic coverage of the collector on the country level. However, it can be set to any topology level (e.g. Carpathians, Caucasus, Alps, Mediterranean, Europe, Middle Asia etc.). The taxonomy ‘area’ is used to display the area of interest and/or specialisation of the collector in the sense of plant systematics. For the Collectors of Ukrainian Herbaria, the classification is set at the level of six formal groups: Spermatophytes, Pteridophytes, Bryophytes, Algae, Fungi and Lichens. Such formal groups are applied in IPNI (2026), Harvard Index of Botanists (2026) and JSTOR Global Plants (2026). Therefore, I decided to use them to keep the data as synchronous as possible. Nevertheless, any level of biological taxonomy can be applied or even different levels can be mixed. The taxonomy ‘herbarium’ is used to attribute the collector to a certain herbarium. In the case of Collectors of Ukrainian Herbaria, at the moment, there are five herbaria listed – LWS (principal testing herbarium), LWKS, LW, LWFU and KW (see Thiers (2026) for abbreviations). All three taxonomies are applied to automatically create respective thesauri. For example, if in the field Herbarium, input the new value KW, it will be automatically added to the ‘herbarium’ thesaurus and later will pop up as a suggested value for the next posts. Moreover, the values from the taxonomies ‘geography’, ‘area’ and ‘herbarium’ are further used for searching and filtering in the main database (Fig. 1). Additionally, they are applied to synchronise the collectors with the interactive map and for statistics visualisation.

The expected settings for the CPT UI plugin are provided in the config/cpt-ui-export.txt file in the distributed repository. To apply these settings, go to CPT UI → Tools → Import/Export and copy-paste the code from the config/cpt-ui-export.txt file.

The plugin *Advanced Custom Fields (ACF)* (WP Engine 2026) is used to set up fields that are filled during the creation of the collector profile. These fields can be of different types (e.g. image, text, number, URL etc.) and are subdivided into two field groups: ‘Collector Portfolio’ and ‘Collector Identifiers’. The group ‘Collector Portfolio’ contains 27 (+13 fields stored under the three groups, i.e. Portrait rights, IndExs records and Label rights) fields describing the collector biography, representing the additional notes and references, as well as links to collector's profiles in other databases (Table 1). The group ‘Collector Identifiers’ comprises only three fields (Table 1) and is specially constructed for the issue of LSIDs (Life Science Identifiers; Bafna et al. (2008)) to each collector. There is no need to fill in these three fields when completing the collector’s profile, as they will be completed automatically after saving. These fields correspond to the unique object identifier of the collector within the database (is equal to 6-digit post number, (e.g. 000257), its version (it is automatically set to 1 after saving, for example, 000257-1) and LSID in URN format (e.g. urn:lsid:herbua.com:collectors:000257-1). LSIDs are also resolved and can be used as hyperlinks (e.g. https://wp.herbua.com/lsid/collectors/000257-1). Object identifiers can also be applied as persistent URIs (e.g. https://wp.herbua.com/id/collectors/000257) to reach the collector’s profile with redirection to the original URL (e.g. https://wp.herbua.com/collector/becker-alexander/). The application of LSIDs aims to achieve free, resolvable and persistent identification of collector profiles at the record level and to track their versioning (McMurry et al. 2017).

The expected settings for the ACF plugin are stored in the folder themes/blocksy-child/acf-json/. The file functions.php contains the filters for ACF JSON. Hence, the expected setting should be auto-applied after ACF plugin activation. If this does not appear, open Custom Fields → Field Groups → click Sync (bulk sync if available). If this approach also does not work, the respective ACF groups and fields can be manually created, as outlined in Table 1.

The role of the plugin *WP All Export* (Soflyy 2026) is limited to creating a backup of the collectors’ database. Such a link can be created in any other way, but the WP All Export plugin is handy as it allows you to keep this link stable during updates to the backups. The link to the generated backup has to be placed in the archive-collector.php file in a child theme (the place for the link is indicated within the provided file), which ensures the work of the ‘Download CSV’ button for users. Respective information, along with step-by-step instructions, is also provided in the README.md file.

**Blocksy child theme**

The Blocksy child theme should be uploaded to the folder wp-content/themes/blocksy-child (e.g. using file manager in C-Panel) and, after that, activated through the WordPress interface using the standard procedure (Dashboard → Appearance → Themes → [choose Blocksy Child]).

The search and filtering options are built into the page with the main collectors’ database, which is represented in a table format (Fig. 1). Together with the template of the collector profile pages, it is realised through the specially prepared Blocksy child theme. Blocksy (Creative Themes 2026) has been chosen as it is one of the popular WordPress themes, easy to maintain even for users with little experience. The file archive-collector.php is responsible for the main database appearance, as well as search and filtering, while the file single-collector.php is responsible for the appearance of the collector profile pages. These files are also responsible for interaction with ACF and CPT UI plugins. In fact, the collectors’ database is fully functional with these two plugins alone and the Blocksy child theme is installed. Other plugins play a supportive role, enhancing the website's user-friendliness.

**herbUA Collectors supportive plugins**

Custom plugins should be uploaded to the wp-content/plugins/folder (e.g. using the file manager in C-Panel) and then activated in the WordPress interface's plugins menu using the standard procedure (Dashboard → Plugins → Installed plugins → [choose required plugin] → Activate). These custom plugins can be applied using shortcodes anywhere on the website and can be represented as separate webpages (e.g. statistics and map) or be part of a complex webpage (e.g. slider). Examples of the recommended shortcodes (e.g. [collector_portraits_slider width="150" ratio="3/4" radius="12" autoplay="yes" speed="13000" pause="yes" reverse="no" count="200"]) are provided in the respective README.md files.

**Figure 2.**
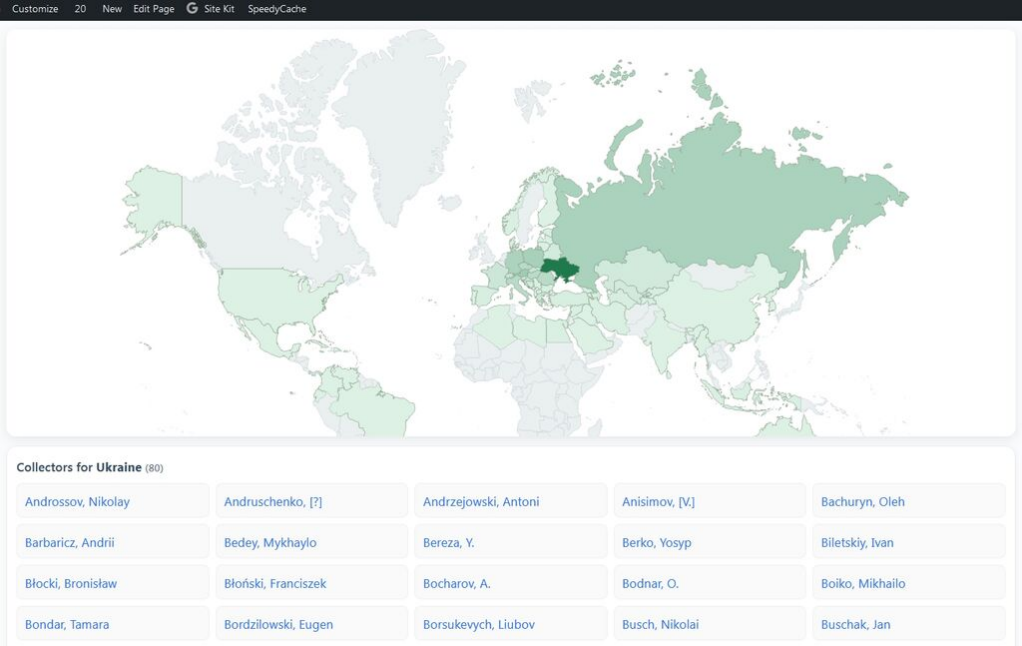
The plugin of the Interactive Map applied to the Collectors of Ukrainian Herbaria website.

The plugin of the *Interactive Map* (collector-country-svg-map.php) is realised through an in-built world map in SVG format (MapSVG 2026). It allows you to visualise the scalable map and use it to look up collectors from specific countries. The number of collectors per country is reflected by the colour gradient (Fig. 2).

**Figure 3.**
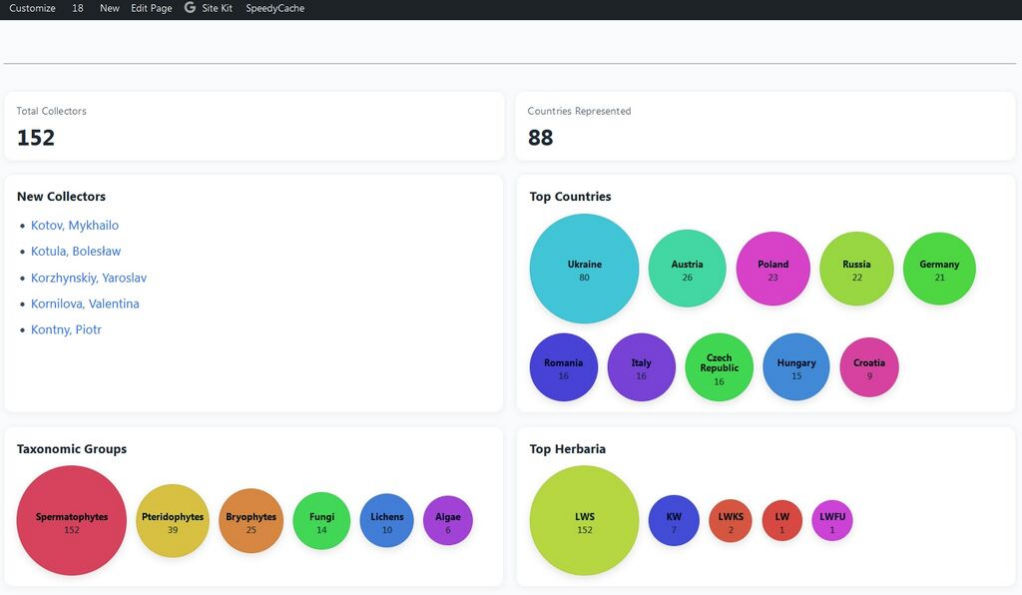
The plugin of the General Statistics applied to the Collectors of Ukrainian Herbaria website.

*General Statistics* plugin serves for visualising the general collectors-related statistics (Fig. 3), including:

- the total number of databased collectors;
- the number of represented countries;
- the bubble plot of the top ten countries by the number of represented collectors;
- the bubble plot of the top ten herbaria by the number of represented collectors;
- the bubble plot showing the number of collectors represented per different taxonomic groups (i.e. Spermatophytes, Pteridophytes, Bryophytes, Algae, Fungi and Lichens);
- the top five collectors recently added to the database.

The number of displayed names, top herbaria and top countries, as well as the appearance of the bubble plots, can be modified in the collector-overview.php file.

**Figure 4.**
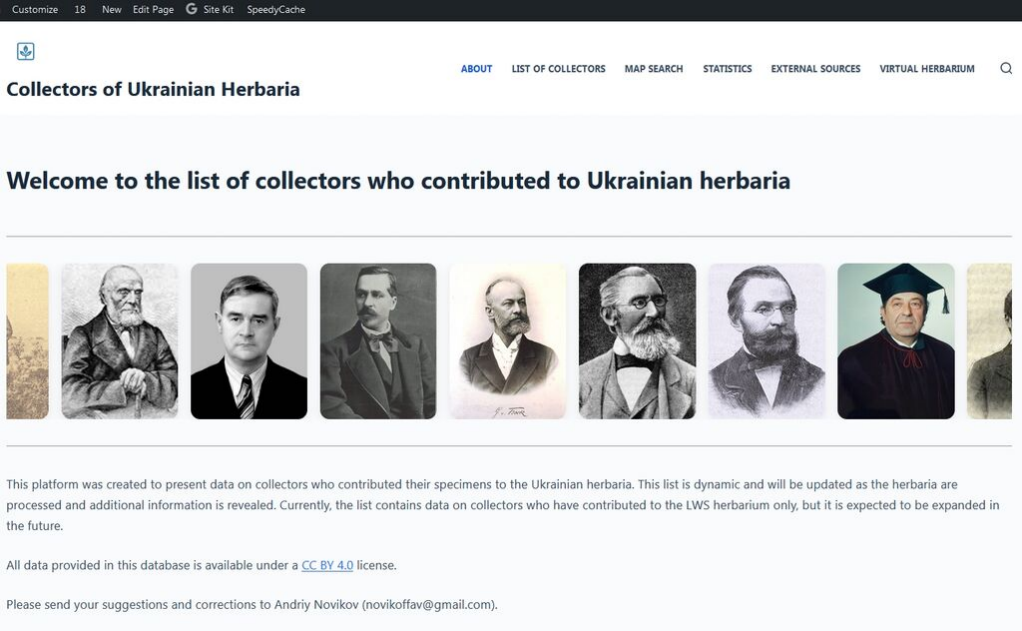
The plugin of the Portraits Slider applied to the Collectors of Ukrainian Herbaria website.

The *Portraits Slider* plugin (collector-portraits-slider.php) is used to visualise the portraits of the collectors (Fig. 4). It is configured to ignore the profiles without portraits. Files man_avatar.png, woman_avatar.png and institution_avatar.png are provided in the distributed repository and are used to filter out such profiles.

**Installation procedure**

1. Install WordPress and log in to wp-admin:

2. Install Blocksy theme (parent theme), then activate it:

3. Install and activate the next plugins through WordPress (free versions are enough):

- ACF (Advanced Custom Fields);
- CPT UI (Custom Post Type UI);
- WP All Export.

4. Copy the repo contents:

- plugins/* → wp-content/plugins/;
- themes/blocksy-child → wp-content/themes/blocksy-child;

5. In wp-admin:

- Activate the blocksy-child theme;
- Activate three custom plugins (i.e., collector-overview, collector-country-svg-map, and collector-portraits-slider);
- Configure CPT UI plugin;
- Configure ACF plugin (if the fields were not auto-applied).

**Download and data access options**

There are two main options for data: (a) bulk download using the ‘Download CSV’ button on the page of the main database; (b) custom download using the inbuilt API facilities. The following rules can be applied to access the data using the built-in WordPress API:

- *List of all collectors*: https://YOURDOMAIN/wp-json/wp/v2/collector?per_page=100&page=1. For example, https://wp.herbua.com/wp-json/wp/v2/collector?per_page=100&page=1. Pagination note: per_page maximum value is 100;
- *Single collector by ID*: https://YOURDOMAIN/wp-json/wp/v2/collector/{ID}. For example, https://wp.herbua.com/wp-json/wp/v2/collector/000986;
- *Search by text (title, content)*: https://YOURDOMAIN/wp-json/wp/v2/collector?search=FREE_TEXT. For example, https://wp.herbua.com/wp-json/wp/v2/collector?search=Kolischuk;
- *Filters by taxonomy*:

- by geodetic term ID(s): ?geographic_coverage=SVG_ID_1 or ?geographic_coverage=SVG_ID_1,SVG_ID_2. For example, https://wp.herbua.com/wp-json/wp/v2/collector?geographic_coverage=UA&per_page=100&page=1;

- by slug(s): ?geographic_coverage_slug=COUNTRY. For example, https://wp.herbua.com/wp-json/wp/v2/collector?geographic_coverage=Ukraine&per_page=100&page=1.

Additionally, customised data can be accessed via the specially developed API:

- *List of all collectors*: https://YOURDOMAIN/id/collectors.json or https://YOURDOMAIN/id/collectors.json?per_page=100&page=1. For example, https://wp.herbua.com/id/collectors.json or https://wp.herbua.com/id/collectors.json?per_page=100&page=1;
- *Single collector by ID*: https://YOURDOMAIN/id/collectors/{ID}?format=json or https://YOURDOMAIN/id/collectors/{ID}.json. For example, https://wp.herbua.com/id/collectors/000257?format=json or https://wp.herbua.com/id/collectors/000257.json.

**Development perspectives**

Further development of the herbUA Collectors framework will include advanced search and filtering options, such as the years of life and collecting activity, a bulk export option and integration with the virtual herbarium. An automatic archiving strategy should also be implemented, as it is currently handled manually by depositing a CSV dump and herbarium labels images in Zenodo (Novikov 2026). At the same time, the Collectors of Ukrainian Herbaria will be continuously updated with new collector profiles and extended to other Ukrainian herbaria.

## Web location (URIs)

Homepage: https://wp.herbua.com/

Download page: https://github.com/novikoffav/herbua-wp-custom

## Technical specification

Platform: WordPress 6.9

Programming language: PHP

Operational system: Linux, Windows, macOS

## Repository

Type: Git

Browse URI: https://github.com/novikoffav/herbua-wp-custom

## Usage licence

### Usage licence

Other

### IP rights notes

GNU General Public License v3.0

## Additional information

### Afterword

The herbUA Collectors framework was developed by me, Andriy Novikov, who is the curator of the LWS Herbarium. Unfortunately, I am not a professional coder, but rather a botanist with a passion for data. So this framework could be imperfect. However, I pursued the general idea of helping other curators, like myself and making their work easier. Therefore, please feel free to send me any suggestions or questions you may have and I will be happy to assist. If you need help during the installation of this framework, I will be glad to assist you.

## Figures and Tables

**Table 1.** Field types are applied to construct the collector's profile. Note: Five fields of label examples can be replaced by one field of the type Gallery in the paid version of ACF. Similarly, five fields in the IndExs group can be replaced by a multiplier in the paid version of the ACF plugin. Choices for selective fields: * – cc : Creative Commons; public_domain : Public domain; permission : Used with permission; copyrighted : Copyrighted / All rights reserved; unknown : Unknown. ** – CC_BY : CC BY; CC_BY_SA : CC BY-SA; CC_BY_NC : CC BY-NC; CC_BY_ND : CC BY-ND CC0. *** – CC_BY : CC BY; CC_BY_NC : CC BY-NC; CC_BY_SA : CC BY-SA; CC_BY_ND : CC BY-ND; CC0 Public_Domain : Public Domain; All_Rights_Reserved : All rights reserved.

Nr	Label	Name	Type
	**Collector Portfolio**		
1	Portrait	portrait	Image
2	Portrait rights	portrait_rights	Group
	Rights type *	rights_type	Select
	Credit	credit	Text
	Source URL	source_url	URL
	CC license **	cc_license	Select
	License URL	license_url	URL
3	Surname	surname	Text
4	Name	name	Text
5	Standard form	standard_form	Text
6	Alternative names	alternative_names	Text
7	Living years	living_years	Text
8	Activity years	activity_years	Text
9	ORCID	orcid	Link
10	Bionomia	bionomia	Link
11	Wikipedia	wikipedia	Link
12	Wikidata	wikidata	Link
13	IPNI	ipni	Link
14	VIAF	viaf	Link
15	HUH	huh	Link
16	Zobodat	zobodat	Link
17	JSTOR Global Plants	jstor	Link
18	IndExs records	indexs_group	Group
	IndExs_1	indexs_1	Link
	IndExs_2	indexs_2	Link
	IndExs_3	indexs_3	Link
	IndExs_4	indexs_4	Link
	IndExs_5	indexs_5	Link
19	Biography	biography	Text Area
20	Notes	notes	Text
21	References	references	Text
22	Label example	label_example	Image
23	Label example 2	label_example_2	Image
24	Label example 3	label_example_3	Image
25	Label example 4	label_example_4	Image
26	Label example 5	label_example_5	Image
27	Label rights	label_rights	Group
	License ***	license	Select
	Attribution	attribution	Text
	Source URL	source_url	URL
	**Collector Identifiers (LSID)**		
1	HerbUA Object ID	herbua_object_id	Text
2	HerbUA Version	herbua_version	Number
3	HerbUA LSID	herbua_lsid	Text
